# Progesterone receptor positivity is a predictor of long-term benefit from adjuvant tamoxifen treatment of estrogen receptor positive breast cancer

**DOI:** 10.1007/s10549-016-4007-5

**Published:** 2016-10-08

**Authors:** Anna Nordenskjöld, Helena Fohlin, Tommy Fornander, Britta Löfdahl, Lambert Skoog, Olle Stål

**Affiliations:** 1 Department of Oncology, Institute of Clinical Sciences, Sahlgrenska Academy, Gothenburg, Sweden; 2 Department of Medicine, Southern Älvsborg Hospital, Borås, Sweden; 3 Department of Clinical and Experimental Medicine, Division of Oncology, Linköping University, Linköping, Sweden; 4 Department of Clinical and Experimental Medicine, Regional Cancer Center Southeast Sweden, Linköping University, 581 85 Linköping, Sweden; 5 Department of Oncology, Karolinska University Hospital, Stockholm, Sweden; 6 Department of Oncology and Pathology, Karolinska Institute, Stockholm, Sweden; 7 Regional Cancer Center Stockholm-Gotland, Stockholm, Sweden; 8 Department of Pathology, Unilabs, St Göran Hospital, Stockholm, Sweden; 9 Department of Pathology and Cytology, Karolinska University Hospital, Stockholm, Sweden

**Keywords:** Breast cancer, Tamoxifen, Estrogen receptor, Progesterone receptor

## Abstract

**Purpose:**

The independent predictive information from progesterone receptor (PgR) positivity for breast cancer treated with tamoxifen has been questioned after an overview by the Early Breast Cancer Trialists’ Collaborative Group (EBCTCG). However, the studies in the overview were to a large content performed before modern PgR immunohistochemistry (IHC) was developed. We therefore investigated the predictive value of PgR determined with IHC in estrogen receptor (ER)-positive tumors from patients participating in the Stockholm trial of adjuvant tamoxifen therapy.

**Methods:**

The Stockholm Breast Cancer Study Group conducted a randomized trial during 1976 through 1990 comparing adjuvant tamoxifen versus control. The patients were stratified according to tumor size and lymph node status in high-risk and low-risk groups. In this study, we evaluated 618 patients with ER-positive “low-risk” breast cancer (size ≤ 30 mm, lymph node-negative) for whom PgR was determined by IHC at one pathology laboratory. The median time of follow-up was 21 years.

**Results:**

Patients with ER-positive tumors that were also PgR-positive by IHC did benefit from tamoxifen, while we could not show any long-term benefit for those with tumors positive for ER only (recurrence rate ratio 0.43, 95 % CI 0.29–0.62 and 0.87, 95 % CI 0.52–1.46, respectively). We further investigated the influence of different levels of PgR positivity on recurrence risk. The results show that at all receptor levels with ≥10 % stained PgR-positive cells, the patients did benefit from tamoxifen. There was no clear linear trend in benefit with increasing proportion of stained cells.

**Conclusions:**

PgR positivity determined by IHC is a marker indicating long-term benefit from adjuvant tamoxifen in patients with ER-positive tumors.

**Electronic supplementary material:**

The online version of this article (doi:10.1007/s10549-016-4007-5) contains supplementary material, which is available to authorized users.

## Introduction

The estrogen and progesterone receptors are predictors of the benefit of endocrine therapy in both primary and metastatic breast cancers [1–4]. Before approximately 1995, cytosol ER and PgR were measured by ligand binding or immunochemical methods measuring receptor content in tumor tissue consisting of both cancer cells and stromal cells. With immunohistochemistry (IHC), which does not require fresh material, ER and PgR are assessed in cancer cells only. Comparing different levels of hormone receptors in relation to the efficacy of adjuvant tamoxifen, the EBCTCG was unable to find a predictive value of PgR in patients known to have ER-positive disease [1]. This is in contrast to findings with adjuvant tamoxifen therapy of premenopausal patients demonstrating PgR determined by IHC to be a stronger predictor of tamoxifen benefit than ER [3]. The aim of this study was to investigate the predictive value of PgR determined by IHC in ER-positive breast cancer. A second aim was to investigate if the effect varies over time and/or with increased levels of PgR positivity. For this, we used tumors from a well-defined randomized clinical adjuvant trial with long-term follow-up. We further discuss whether the method of assessing the hormone receptors may affect trial conclusions.

## Methods

### Study design

**Fig. 1:**
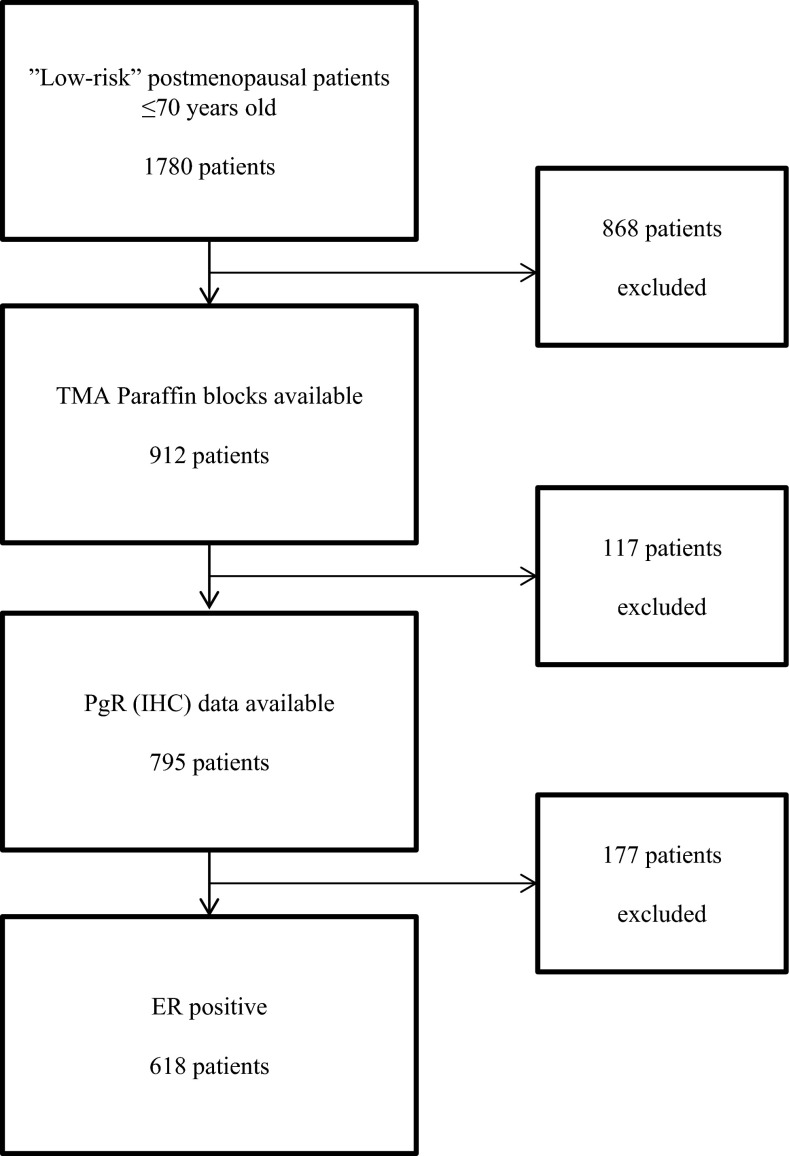
Summary of trial design

Patients with operable invasive breast cancer were entered previously in detail described study of adjuvant tamoxifen therapy conducted by the Stockholm Breast Cancer Study Group [4]. Postmenopausal women younger than 70 years were randomly given tamoxifen postoperatively at a dose of 40 mg per day compared with no adjuvant endocrine therapy. During November 1976 through June 1990, 2738 patients entered the trial. Among them, 1780 patients (65 %) with no lymph node metastases and a tumor diameter of 30 mm or less (established by histological examination) were classified as “low risk” and did not receive cytotoxic chemotherapy. In this group, 432 patients were treated with breast conserving surgery including axillary dissection plus radiation to the breast (50 Gy/5 weeks). The remaining 1348 patients had a modified radical mastectomy and no radiotherapy. From the low-risk patients, we found paraffin blocks from 912 for construction of microtissue arrays (TMAs). The trial included patients irrespectively of hormone receptor content, but prospectively collected data on ER and PgR status were available and archived tumor tissue had sufficiently high quality for IHC analysis in 795 cases. These patients had similar age distribution, tumors of similar size, and proportion of ER-positive tumors as the entire group of 1780 patients with low-risk tumors. The proportion of patients randomized to tamoxifen therapy was 52 % as compared to 50 % in the entire group. Among the tumors analyzed for PgR by IHC, 591 were ER-positive as determined by IHC, while 27 tumors with missing data on ER by IHC were ER-positive according to cytosol analysis, resulting in 618 ER-positive tumors (Fig. 1).

### Follow-up strategies

Yearly clinical visits included a physical examination and a mammogram. Chest X-ray, blood sampling, bone scans, etc., were performed if clinical symptoms and signs indicated a probable relapse. Disease recurrence was confirmed when possible by biopsy. Recurrence was dated from the first evidence of relapse based on physical, histological, or imaging data. After recurrence, treatment was decided individually. The current results were based on follow-up until December 31, 2014.

### Hormone receptor determination

Before 1988, ER and PgR were determined using isoelectric focusing on polyacrylamide gel as previously described [5]. After 1988, an enzyme immunoassay was used. For ER, studies have shown that results with these techniques correlate well with those obtained using methods based on dextran-coated charcoal and IHC [2]. The cytosol receptor values were normalized to DNA content, and a receptor content of ≥0.05 fmol/µg DNA was classified as positive. The IHC staining was performed using the Ventana HX automatic system BenchMark (Ventana Medical System, SA IllKirch, Cedex, France). Primary monoclonal antibodies were the CONFIRM™ mouse anti-ER antibody (clone 6F11) and the CONFIRM™ mouse anti-PR antibody (clone 16) from Ventana Medical Systems. Antigen retrieval and staining procedure were performed according to the instruction by the Ventana manufacture. Positive controls were run with each batch. Only the invasive part of the carcinoma was assessed, and for each case, all three cores of the TMA were reviewed. The receptor levels presented are based on an average of the three cores of the TMA. The proportion of stained nuclei was recorded as 0, 1–9 %, 10–24 %, 25–49 %, 50–74 %, 75–89 %, and ≥90 %. The scoring was done by two pathologists (B.L; L.S.).

### Statistical methods

To compare the association between PgR expression and clinical parameters, the Pearson Chi squared test (categorical variables) and the Student’s *t* test (continuous variables) were applied. Time for follow-up was defined as the time from randomization until the first event, death, or last observation. For cumulative recurrence risk (CRR) and cumulative distant recurrence risk (CDRR), the last observation was December 31, 2014 and for cumulative breast cancer-specific mortality (CBCSM) December 31, 2012. CRR, CDR,R and CBCSM were estimated by the Kaplan–Meier method. The events in calculations of CRR were loco-regional recurrence, distant recurrence, and death due to breast cancer. In the calculations of CDRR, the events were distant recurrence and death due to breast cancer, and in CBCSM death due to breast cancer. In all these analyses, we censored for death due to other causes. Hazard ratios (HR) and 95 % confidence intervals (CIs) were estimated using the Cox’s proportional hazards model. A *p* value of <0.05 was considered to be statistically significant. The proportional hazards assumption was checked by log-minus-log plots of the hazard functions. In analyses where the proportional hazards assumptions was violated, Cox regression divided by different time periods was applied. We also examined crude cumulative incidence rates [6]. This is failure probabilities for a particular type of event, in the presence of other events, which may impede the event of interest to occur. Death due to other causes than breast cancer was considered as a competing event. In order to understand the pattern of the treatment effect of tamoxifen for different PgR values, a subpopulation treatment effect pattern plot (STEPP) analysis was performed [7]. For this purpose, the program *stepp tail* implemented in Stata was used [8]. The parameter *g* was set to 7, generating 13 overlapping subgroups (six for decreasing PgR values, one for all patients, and six for increasing PgR values). The STEPP figures show the estimated effect of tamoxifen in each of these subgroups from a graphical view.

The statistical analyses were performed using STATA/SE 13.1. Our study was reported according to the Reporting Recommendations for Tumor Marker Prognostic Studies (REMARK) criteria [9].

## Results

The PgR expression was analyzed with IHC for 795 tumors. Almost half of them (375 tumors) were considered as PgR negative with <10 % stained cells. Furthermore, the percentage of PgR-positive tumor cells was as follows: 10–49 % in 127 tumors, 50–74 % in 119 tumors, and ≥75 % in 174 tumors. Table 1 shows other tumor characteristics in relation to PgR status. It is notable that nearly all PgR-positive tumors were also ER-positive and seldom HER2-positive.

**Table 1:** Characteristics for patients with PgR expression determined by IHC

	PgR n (%)		Total	*p* value
	<10 %	≥10 %		
Total no. of patients	375	420	795	
*Age*				
Median (IQR)	62 (58–66)	63 (58–67)	62 (58–67)	0.41
*Tumor size (mm)*				
<20	225 (61)	307 (74)	532 (68)	<0.001
≥20	144 (39)	107 (26)	251 (32)	
Unknown	6	6	12	
*ER status*				
Negative	161 (43)	9 (2)	170 (22)	<0.001
Positive	211 (57)	407 (98)	618 (78)	
Unknown	3	4	7	
*PgR status (cytosol)*				
Negative	205 (78)	92 (30)	297 (52)	<0.001
Positive	57 (22)	214 (70)	271 (48)	
Unknown	113	114	227	
*HER2* ^a^				
Negative	280 (80)	388 (97)	668 (89)	<0.001
Positive	72 (20)	13 (3)	85 (11)	
Unknown	23	19	42	

^a^HER2 was assessed with immunohistochemistry as previously described [23]

### Effect of tamoxifen in subgroups

Results from the present trial have previously shown a significantly reduced recurrence rate among patients with ER-positive tumors randomized to tamoxifen therapy versus control [*HR* = 0.53 (0.37–0.74), *p* < 0.001] [2].

Patients with ER+/PgR+ tumors receiving tamoxifen had a reduced recurrence risk compared with those who were not treated with tamoxifen (*HR* = 0.43, 95 % CI 0.29–0.62, *p* < 0.001) (Table 2). For patients with ER+/PgR− tumors, the effect of the treatment was time-dependent. The first 5 years after diagnosis the tamoxifen-treated patients had a reduced recurrence risk (*HR* = 0.39, 95 % CI 0.15–1.00, *p* = 0.05), whereas it increased thereafter (*HR* = 1.34, 95 % *CI* 0.69–2.60, *p* = 0.39) (Table 3). P for interaction between PgR status and treatment was 0.55 the first 5 years and 0.03 after this time period. Seen over the whole time period, the relative risk ratio for tamoxifen treatment versus the control group when comparing PgR+ and PgR− tumors was 0.49 (95 % CI 0.25–0.92, *p* = 0.03). During the first 5 years, it was 0.70 (95 % CI 0.22–2.22, *p* = 0.55) and during the latter time period 0.41 (95 % CI 0.18–0.92, *p* = 0.03).

**Table 2:** Outcome for patients with ER positive tumors divided by PgR (IHC) status

Tam vs. control	PgR (IHC)	Number of patients/events		HR (95 % CI)	*p* value	P for interaction
		TAM	Control			
Recurrence rate	All	329/70	289/102	0.54 (0.40–0.74)	<0.001	0.03
	≥10 %	225/43	182/72	0.43 (0.29–0.62)	<0.001	
	<10 %	104/27	107/30	0.87 (0.52–1.46)	0.59	
Distant recurrence rate	All	329/51	289/79	0.54 (0.38–0.76)	0.001	0.17
	≥10 %	225/31	182/53	0.45 (0.29–0.70)	<0.001	
	<10 %	104/20	107/26	0.75 (0.42–1.34)	0.33	
Breast cancer specific mortality rate	All	329/43	289/72	0.51 (0.35–0.74)	<0.001	0.33
	≥10 %	225/26	182/47	0.44 (0.27–0.71)	0.001	
	<10 %	104/17	107/25	0.65 (0.35–1.21)	0.17

**Table 3:** Outcome for patients with ER positive tumors divided by PgR (IHC) status and different time periods

Tam vs. control	PgR (IHC)	0–5 years				Beyond 5 years			
		Number of patients/events		HR (95 % CI)	*p* value	Number of patients/events		HR (95 % CI)	*p* value
		TAM	Control			TAM	Control		
Recurrence rate	All	329/18	289/48	0.31 (0.18–0.53)	<0.001	294/52	228/54	0.74 (0.51–1.09)	0.13
	≥10 %	225/12	182/33	0.27 (0.14–0.53)	<0.001	199/31	143/39	0.55 (0.34–0.88)	0.012
	<10 %	104/6	107/15	0.39 (0.15–1.00)	0.051	95/21	85/15	1.34 (0.69–2.60)	0.39
Breast cancer specific mortality rate	All	329/7	289/14	0.43 (0.18–1.08)	0.072	305/36	265/58	0.53 (0.35–0.80)	0.002
	≥10 %	225/3	182/9	0.27 (0.07–0.99)	0.049	208/23	169/38	0.48 (0.29–0.81)	0.006
	<10 %	104/4	107/5	0.81 (0.22–3.00)	0.75	97/13	96/20	0.61 (0.31–1.24)	0.17

The risk reduction for the tamoxifen-treated patients vs. the control group was similar considering breast cancer-specific mortality and distant recurrence risk (Table 2). Hazard ratio for breast cancer-specific mortality was 0.44 (95 % CI 0.27–0.71, *p* = 0.001) for ER+/PgR+ patients and 0.65 (95 % CI 0.35–1.21, *p* = 0.17) for ER+/PgR− patients. The corresponding HR for distant recurrence risk was 0.45 (95 % CI 0.29–0.70, *p* < 0.001) for ER +/PgR + patients and 0.75 (95 % CI 0.42–1.34, *p* = 0.33) for ER+/PgR− patients.

The cumulative incidence functions adjusting for competing risks showed lower recurrence risks for both tamoxifen-treated and untreated patients compared with the risks computed with the Kaplan–Meier method (data not shown). However, the relative risks between the treatment groups were similar, irrespective of the statistical method used.

**Fig. 2:**
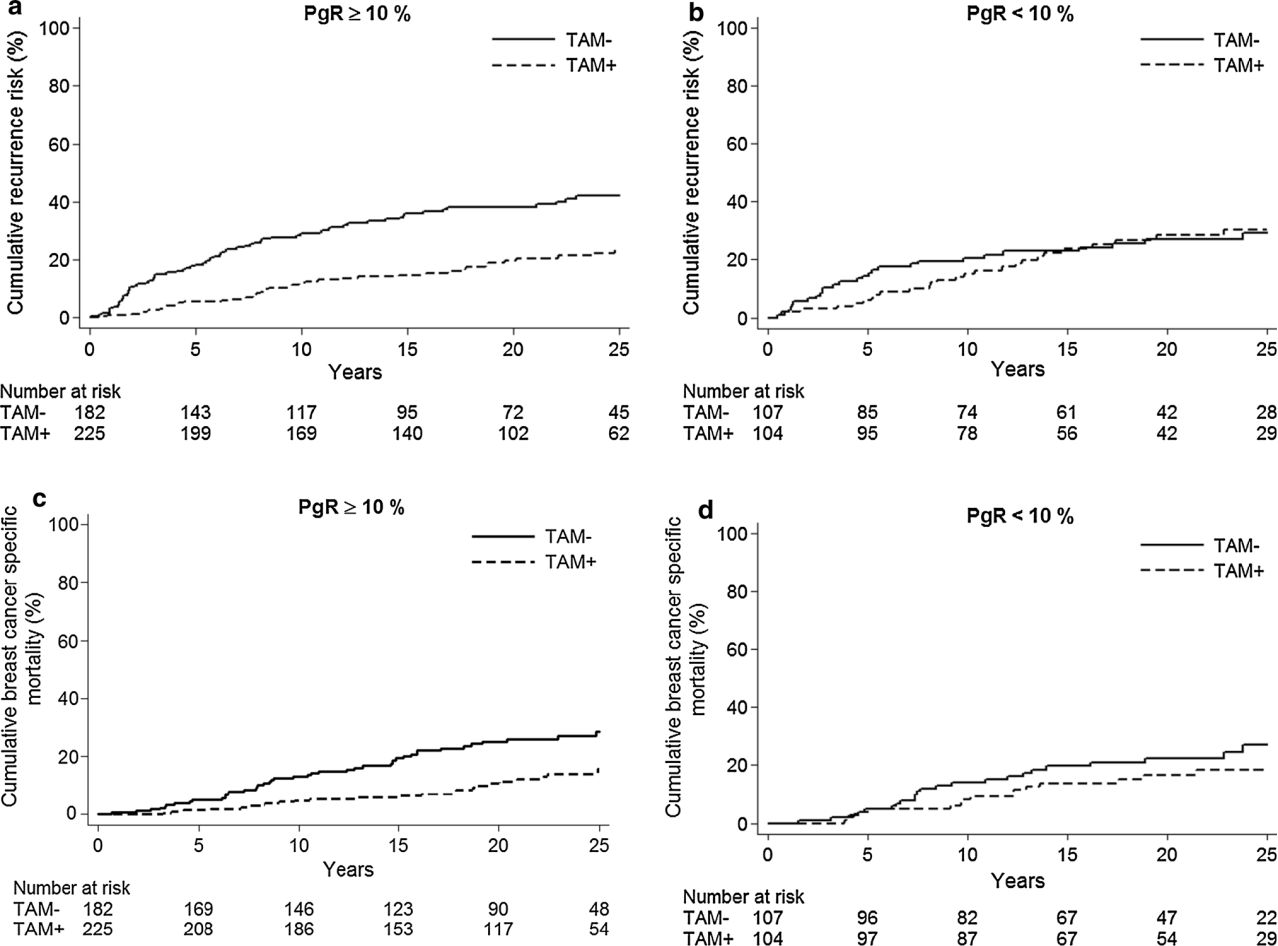
PgR expression determined with IHC. Cumulative recurrence risk for patients with ER+/PgR+ tumors, *HR* = 0.43 (95 % CI 0.29–0.62), *p* < 0.001 (**a**) and ER+/PgR− tumors, *HR* = 0.87 (95 % CI 0.52–1.46), *p* = 0.59 (**b**). Cumulative breast cancer-specific mortality for patients with ER+/PgR+ tumors, *HR* = 0.44 (95 % CI 0.27–0.71), *p* = 0.001 (**c**) and ER+/PgR− tumors, *HR* = 0.65 (95 % CI 0.35–1.21), *p* = 0.17 (**d**)

The results show that tamoxifen therapy resulted in a marked benefit for patients with tumors positive for both receptors, while we could not show any long-term benefit from tamoxifen for those with tumors positive for ER only (Fig. 2; Table 3).

### Effect of tamoxifen at different levels of PgR positivity

**Fig. 3:**
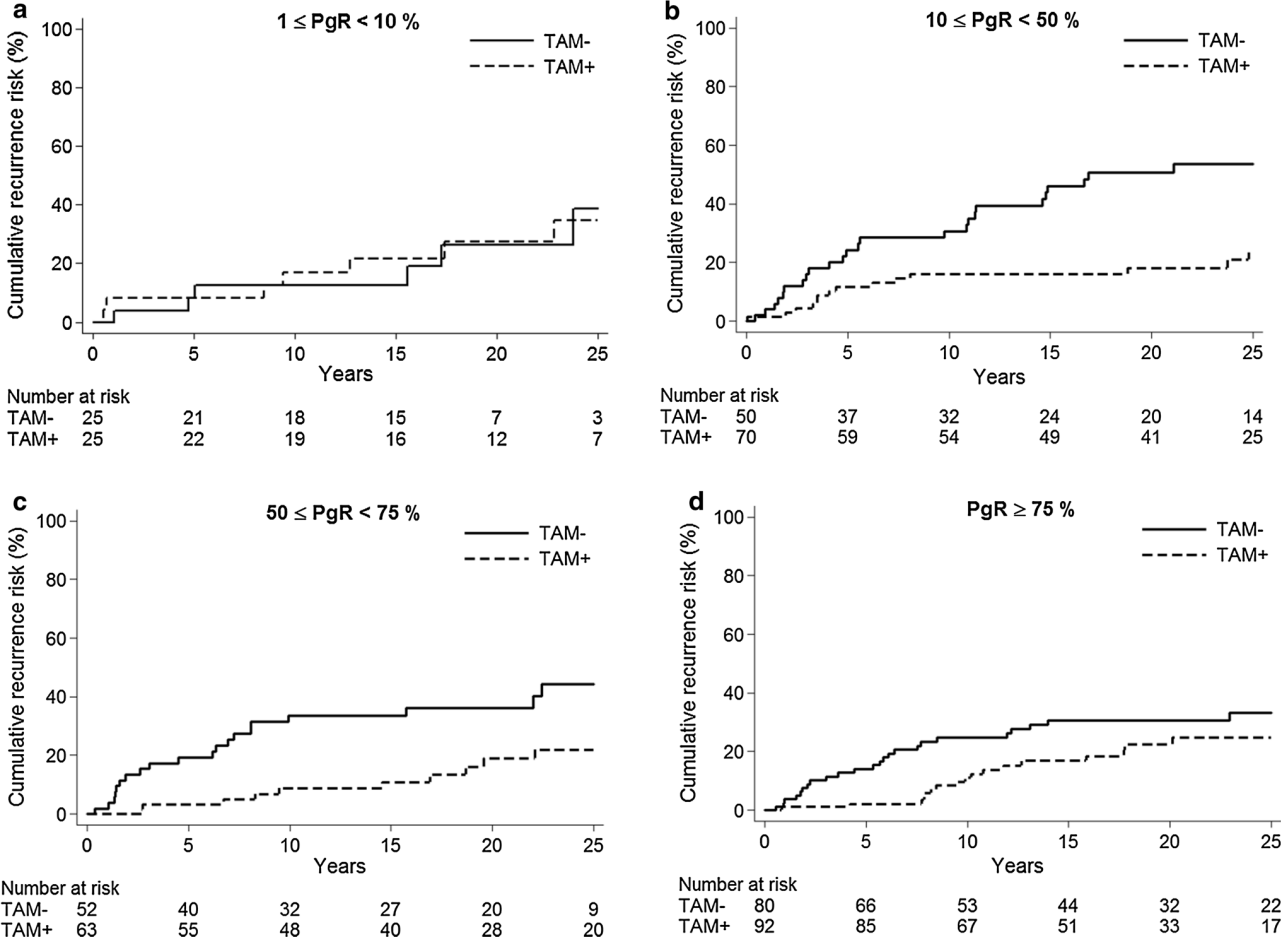
Influence of increasing proportion of PgR-positive tumor cells on cumulative recurrence risk among patients with ER-positive tumors. PgR 1–9 %, *HR* = 1.11 (95 % CI 0.38–3.24), *p* = 0.84 (**a**), PgR 10–49 %, *HR* = 0.30 (95 % CI 0.16–0.58), *p* < 0.001 (**b**), PgR 50–74 %, *HR* = 0.38 (95 % CI 0.18–0.80), *p* = 0.011 (**c**), PgR ≥ 75 %, *HR* = 0.59 (95 % CI 0.32–1.08), *p* = 0.086 (**d**)

**Fig. 4:**
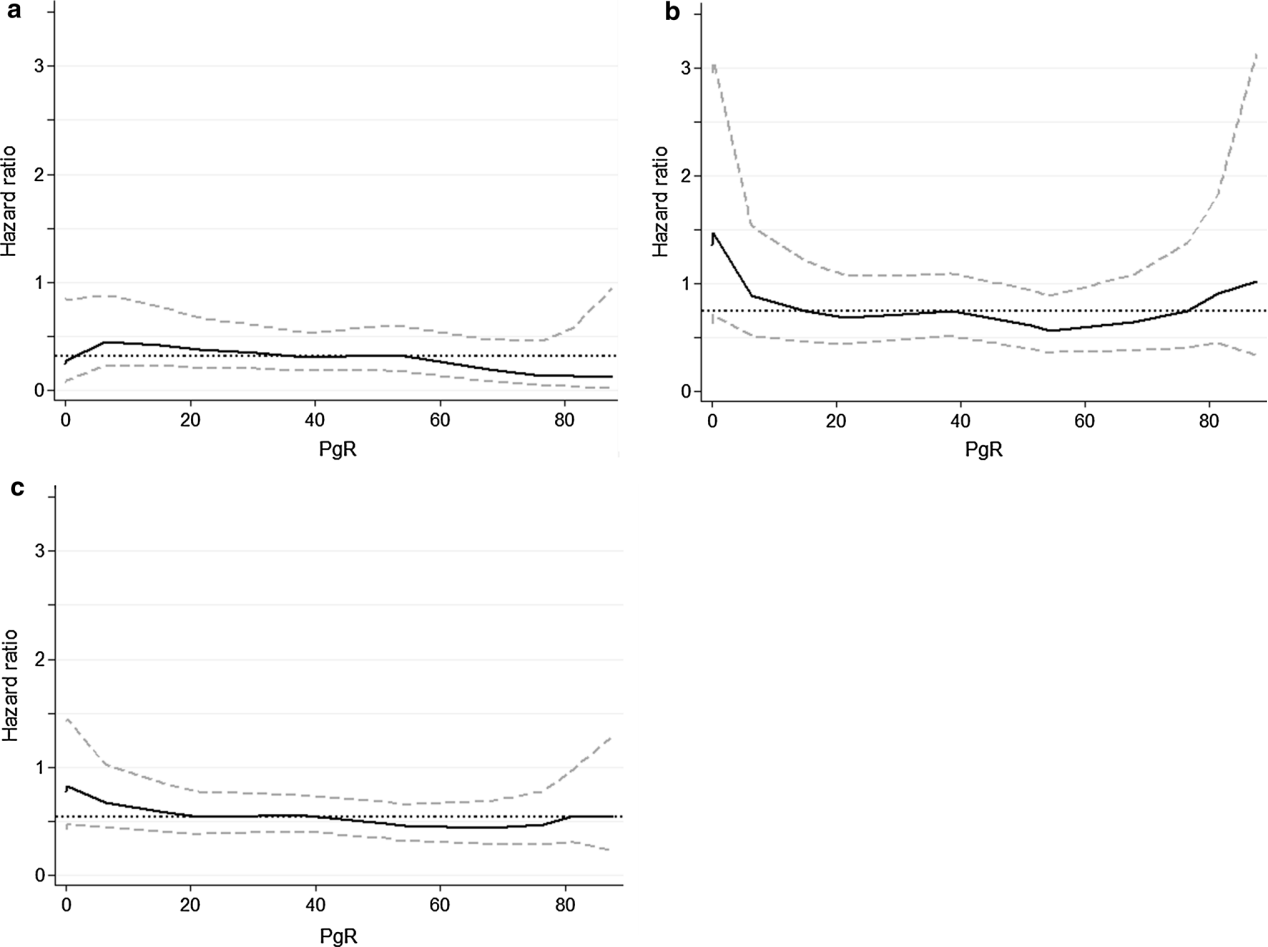
Subpopulation treatment effect pattern plots (STEPP), showing the effect of tamoxifen vs. no tamoxifen on the recurrence risk during the first 5 years after diagnosis (**a**), more than 5 years after diagnosis (**b**) and over the whole time period (**c**) in relation to PgR values measured with IHC. HR (*solid black line*) with the corresponding 95 % confidence interval (*dashed gray lines*) is plotted against the mean PgR. The *dotted black line* shows the HR for tamoxifen vs. control for all PgR values in the selected time period. The analysis was confined to patients with ER-positive tumors

We further investigated the influence of different levels of PgR positivity on the recurrence risk. The results in Fig. 3 and Table 4 show that at all receptor levels with ≥10 % stained cells, there was a benefit from tamoxifen. The hazard ratios between tamoxifen vs. no tamoxifen for the groups of 10–49 %, 50–74 %, and ≥75 % PgR stained cells were 0.30 (95 % CI 0.16–0.58, *p* < 0.001), 0.38 (95 % CI 0.18–0.80, *p* = 0.011), and 0.59 (95 % CI 0.32–1.08, *p* = 0.09), respectively. We could not show any benefit from tamoxifen among patients with 1–9 % PgR-positive tumor cells (*HR* = 1.11, 95 % CI 0.38–3.24, *p* = 0.84). To further investigate tamoxifen treatment effect differences across the continuum of PgR levels, we performed STEPP analyses (Fig. 4). The STEPP curves show the effect of tamoxifen on the recurrence risk during the first 5 years after diagnosis (Fig. 4a), after the first 5 years (Fig. 4b), and over the whole time period (Fig. 4c), for overlapping subgroups with different mean PgR IHC values. The STEPP curves indicated some effect of tamoxifen even in patients with ER+/PgR− tumors the first 5 years after diagnosis. This is in line with the data in Fig. 2 indicating a minor benefit from tamoxifen also for patients with ER-positive tumors with no or less than 10 % PgR− positive cells.

**Table 4:** Outcome for patients with ER positive tumors divided by different levels of PgR (IHC) expression and different time periods

Tam vs. control	PgR (IHC) (%)	0–5 years				Beyond 5 years			
		Number of patients/events		HR (95 % CI)	*p* value	Number of patients/events		HR (95 % CI)	*p* value
		TAM	Control			TAM	Control		
Recurrence rate	0	79/4	82/13	0.29 (0.09–0.89)	0.031	73/15	64/11	1.34 (0.62–2.93)	0.46
	1–9	25/2	25/2	1.07 (0.15–7.56)	0.95	22/6	21/4	1.14 (0.32–4.05)	0.85
	10–49	70/8	50/12	0.44 (0.18–1.07)	0.069	59/6	37/15	0.21 (0.08–0.54)	0.001
	50–74	63/2	52/10	0.15 (0.03–0.69)	0.015	55/9	40/10	0.59 (0.24–1.46)	0.26
	≥75	92/2	80/11	0.15 (0.03–0.67)	0.013	85/16	66/14	0.93 (0.45–1.91)	0.85

**Fig. 5:**
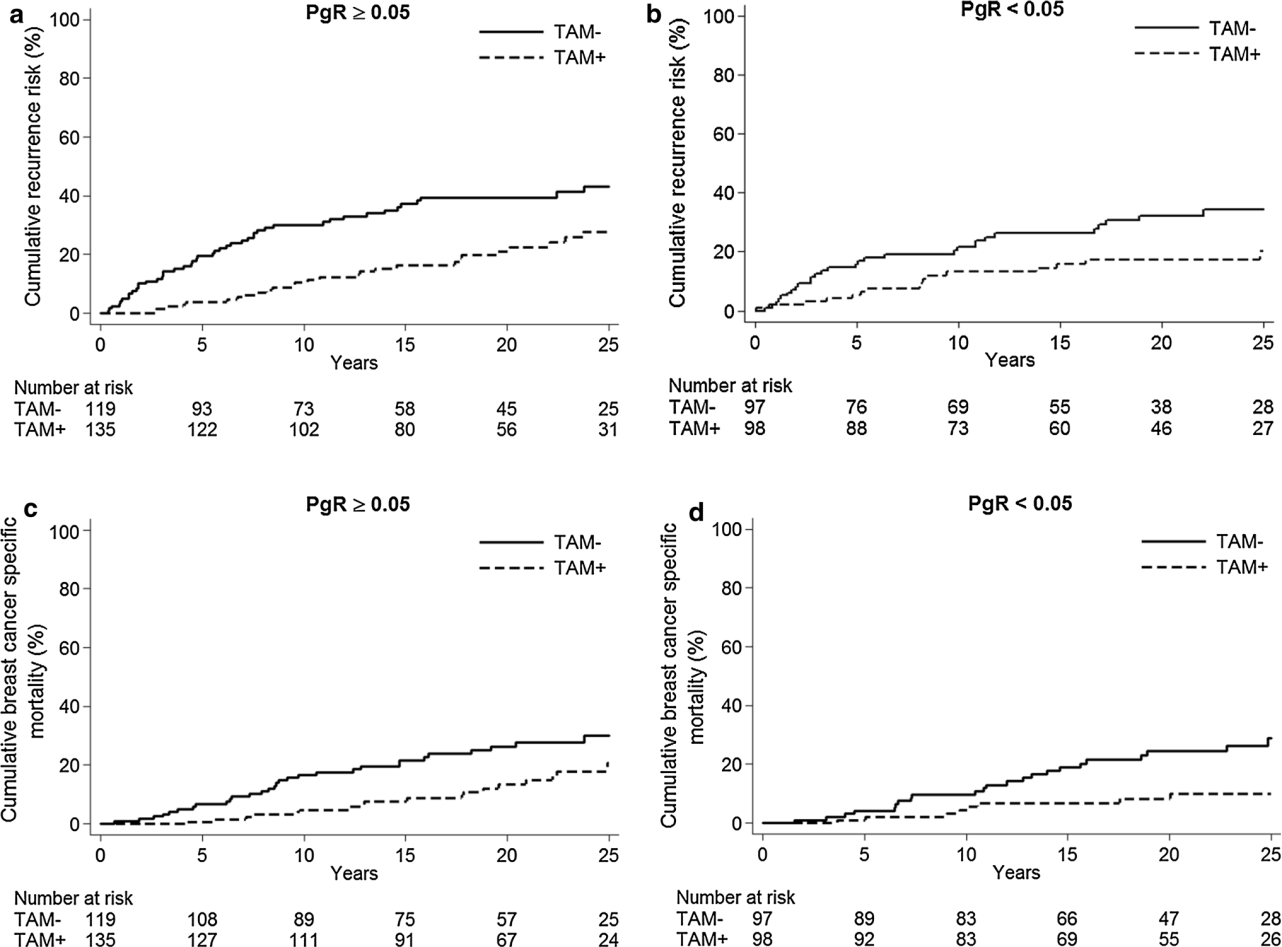
PgR expression determined with cytosol assays. Cumulative recurrence risk for patients with ER+/PgR+ tumors, *HR* = 0.45 (95 % CI 0.28–0.72), *p* = 0.001 (**a**) and ER+/PgR− tumors, *HR* = 0.48 (95 % CI 0.27–0.87), *p* = 0.015 (**b**). Cumulative breast cancer-specific mortality for patients with ER+/PgR+ tumors, *HR* = 0.49 (95 % CI 0.27–0.87), *p* = 0.015 (**c**) and ER+/PgR− tumors, *HR* = 0.35 (95 % CI 0.16–0.75), *p* = 0.007 (**d**)

Our data demonstrating significant more benefit from tamoxifen in patients with tumors positive for both ER and PgR as compared with patients with tumors positive for ER alone, in part contrast to the data in the EBCTCG review. However, as presented in the overview, only a minor proportion of the PgR values were obtained with IHC. In the present cohort of 618 patients with ER-positive tumors, cytosol PgR information was available in 449 cases. In 254 tumors (57 %), the cytosol assay was positive. The data in Fig. 5 and Supplementary Table 1 illustrate that, in agreement with the EBCTCG review, PgR positivity determined with our cytosol assay did not predict tamoxifen benefit more efficiently than ER positivity alone.

## Discussion

We have shown that IHC determined PgR positivity in at least 10 % of the tumor cells predicts reduced recurrence risk after adjuvant tamoxifen therapy for patients with ER-positive tumors. For patients with 1–9 % PgR-positive tumor cells, we observed a similar recurrence rate in both treatment arms. However, this group included only 50 patients with 14 events. We are therefore unable to exclude that this group of patients shows some benefit from tamoxifen.

Our data in part contrast to those in the EBCTCG overview [1]. This discrepancy may be explained by the different techniques used to demonstrate PgR positivity.

In most trials in the EBCTCG overview, different forms of cytosol-based assays were used. It is stated that 21 % of the ER-negative cancers were PgR-positive, indicating that the PgR technique used resulted in many false positive PgR classifications. With modern IHC and gene expression assays, it has been clearly demonstrated that PgR positivity or PgR gene expression is a rare event demonstrated in 1–4 % of ER-negative tumors [10]. Another aspect is the possible time dependence of the ability of PgR to predict the efficacy of adjuvant tamoxifen. The follow-up period in the present study was long and the predictive value for PgR was most evident beyond 5 years of follow-up. Furthermore, the patients in the present study did not receive adjuvant chemotherapy. Potential differences in tamoxifen benefit in relation to PgR levels could possibly be masked by the effect of chemotherapy, in particular in cohorts where the use of chemotherapy is not balanced between patients with ER+/PgR+ and ER+/PgR− tumors.

Our present results may be compared to those obtained with the randomized trial comparing 2 and 5 years of adjuvant tamoxifen therapy, which demonstrated that PgR positivity was a strong predictor of the benefit of 5 years of tamoxifen in patients with ER-positive tumors. The results were probably influenced by the fact that a major proportion of the receptor data were obtained with immunoassays, and the cytosol assays were concentrated to two laboratories participating in a quality control program [11]. In a study by Bardou et al. [12], clinical outcomes of patients in two large databases were analyzed as a function of steroid receptor status. Receptor assays were concentrated to two laboratories and in agreement with our data they concluded that when accurately measured, PgR status is an independent predictive factor for benefit from adjuvant endocrine therapy. The significance of PgR has also been demonstrated in metastatic ER+ breast cancer, where increased levels of PgR improved the response rate to tamoxifen [13]. PgR positivity determined by IHC was previously shown to be a strong predictor of the benefit of adjuvant tamoxifen in premenopausal patients [3, 14]; this finding is now extended to postmenopausal patients.

We and others have demonstrated that ER measured by ligand binding and IHC yield similar proportions of ER-positive tumors, and both types of assays may be used to predict response to tamoxifen therapy [2]. In contrast, even the most experienced laboratories have reported poor correlation between PgR data obtained with IHC and ligand binding assays. Elledge et al. found that 38 % of the tumors, which were PgR negative with ligand binding, were PgR-positive with IHC [15]. A similar number of 30 % was found in the present study (Table 1).

Patients with ER- and PgR-positive tumors have their recurrence rate approximately halved by 2 years of adjuvant tamoxifen therapy. Thus, many experience recurrence in spite of having this tumor pattern. We and others have shown that alterations of the PI3K/Akt/mTOR- growth signaling pathway correlate to resistance to endocrine therapy, partly through regulation of ER activity. This mechanism of resistance seems to operate in ER-positive tumors also strongly positive for PgR. This is in line with our previous observation that in tamoxifen-treated patients, low levels of p-mTOR-2448 combined with ER and PgR positivity predicts a prolonged recurrence-free survival [16].

Our data may be compared with those of Dowsett and colleagues using IHC to analyze tumors from the NATO and CRC trials [17]. In contrast to our data, PgR positivity did not predict further tamoxifen benefit for patients with ER-positive tumors. We are unable to explain this difference but a different scoring system yielding a higher proportion of PgR-positive cells may have contributed to the difference. Also, Dowsett et al. found that 13 % of the ER-negative tumors were PgR-positive, and this subgroup tended to benefit from tamoxifen therapy.

PgR is an ER-regulated protein, and the presence of PgR in the cancer cells has long been considered as a result of activated ER. Therefore, our finding that patients with a tumor containing both ER and PgR did benefit from tamoxifen therapy seems reasonable. However, according to Cui et al. [18], the absence of PgR may not only reflect a lack of ER activity, but rather a hyperactive cross talk between ER and growth factor signaling pathways that downregulate PgR even as they activate other ER functions. Therefore, the authors suggest that estrogen depletion therapy, such as aromatase inhibitors, may be more suited for ER+/PgR− tumors. Other studies have not shown any particular advantage of aromatase inhibitors over tamoxifen for ER+/PgR− tumors compared with other subgroups [19–21]. We suggest that IHC determined tumor content of both ER and PgR should be taken into consideration when breast cancer patients receive postoperative advice. Patients with tumors positive for both receptors may be informed that tamoxifen therapy often provides long-term protection against disease recurrence. For patients with tumors positive for ER only, cytotoxic therapy may be discussed as additional treatment, which is also in line with the St Gallen consensus [22].

## Electronic supplementary material

Below is the link to the electronic supplementary material.

Supplementary material 1 (DOCX 62 kb)
