# Supplementary figures and images for: Progesterone receptor positivity is a predictor of long-term benefit from adjuvant tamoxifen treatment of estrogen receptor positive breast cancer
Source: Breast Cancer Res Treat. 2016 Oct 8;160(2):313–22. doi: 10.1007/s10549-016-4007-5 (PMC5065613; doi:10.1007/s10549-016-4007-5)

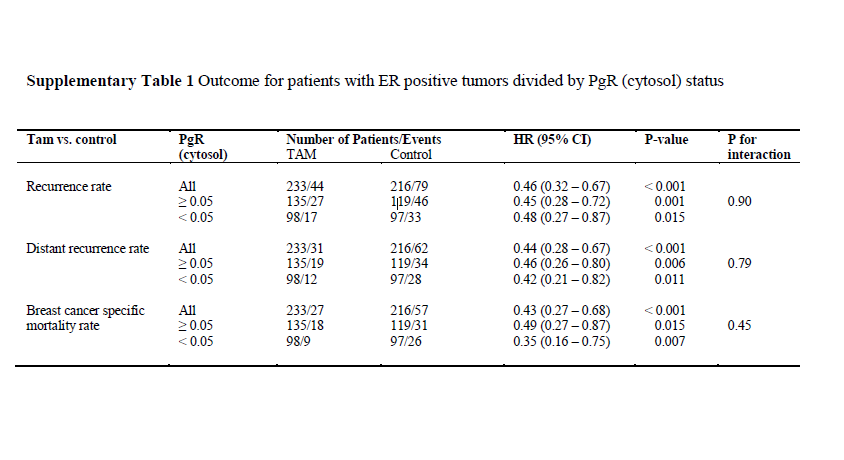

Supplement: Supplementary file 1 — Supplementary material 1 (DOCX 62 kb) [file 10549_2016_4007_MOESM1_ESM.docx]
